# Effect of different exercise training intensities on age-related cardiac damage in male mice

**DOI:** 10.18632/aging.203513

**Published:** 2021-09-14

**Authors:** Zuowei Pei, Chenguang Yang, Ying Guo, Min Dong, Fang Wang

**Affiliations:** 1 Department of Cardiology, Beijing Hospital, National Center of Gerontology, Institute of Geriatric Medicine, Chinese Academy of Medical Sciences, Beijing 100730, China; 2 School of Life Science, University of the Chinese Academy of Sciences, Beijing 100049, China

**Keywords:** aging, cardiac damage, myocardial remodeling, oxidative stress, mice

## Abstract

Aging is the most important risk factor for cardiovascular diseases. Although exercise is known to be beneficial for the health of aging heart, the optimal exercise training intensity to prevent natural aging-induced cardiac damage has not been defined. In this study, we used 32-week-old male mice and randomly divided them into three groups, namely, untrained (UNT) mice, moderate-intensity exercise training (MET) mice, and high-intensity interval training (HIIT) mice. Mice in the two exercise training groups were subjected to exercise 5 days per week for 24 consecutive weeks. Metabolic characteristics, cardiac function and morphology, myocardial remodeling, myocardial fibrosis (collagen III, α-SMA, and TGF-β), oxidative stress (NRF2, HO-1, SOD, and NOX4), and apoptosis (BAX, Bak, Bcl-2, and Bcl-XL) were analyzed 24 weeks after the different treatments. MET improved cardiac function and reduced myocardial remodeling, myocardial fibrosis, and oxidative stress in the aging heart. MET treatment exerted an anti-apoptotic effect in the heart of the aging mice. Importantly, HIIT did not protect against cardiac damage during the natural aging process. These findings suggest that MET may be one of the main methods to prevent cardiac damage induced by natural aging.

## INTRODUCTION

Aging is an important risk factor in the development of cardiovascular diseases [1], probably due to continuous changes in the structure and function of the heart with aging [2]. Aging causes a decrease in cardiac function by reducing cardiac reserve and adverse remodeling. The key molecular phenotypes of cardiac aging include changes in stress response pathways, cardiac energy metabolism, mitochondrial function, cardiomyocyte death, and extracellular matrix remodeling [3–7]. Initially, these changes may be beneficial because they maintain cardiac function, but later, they are usually detrimental to the heart, leading to age-related cardiac remodeling and impaired cardiac reserve, which increases the risk of heart failure and other cardiovascular diseases [8]. As aging increases the risk of cardiovascular diseases and reduces organ functions, studies with a focus on developing interventions against aging-induced cardiovascular diseases to clarify the underlying mechanism may have preclinical and clinical significance.

Exercise training can decrease the risk for cardiovascular diseases and improve cardiac function [9–11]. It also reduces pathological cardiac hypertrophy, which is induced by pressure overload [12]. A previous study showed that exercise training reduces vascular fibrosis in obese rats, but it did not provide evidence that exercise training prevented cardiac damage in naturally aging rats [13]. Exercise training has been shown to decrease aging-induced cardiomyocyte apoptosis, which is the final step in the progression of heart failure, as well as aging-associated long-term stresses including cardiac hypertrophy, inflammation, and fibrosis [14]. Thus, suitable exercise training might prevent heart injury by reducing the progression of heart failure caused by natural aging in rat heart [15, 16]. Although exercise training can reduce the cardiac damage induced by aging, the optimal exercise training intensity to prevent cardiac damage caused by aging remains unknown. A previous study has shown greater improvements in disability and exercise capacity with high-intensity interval training (HIIT) than with moderate-intensity exercise training (MET) in patients with chronic nonspecific low-back pain [17]. In addition, an animal study demonstrated that 3-week HIIT improved post-ischemic functional recovery and reduced infarct size [18]. However, Paolucci et al. found that compared with MET, HIIT not only decreased depressive symptoms such as MET but also increased perceived stress [19]. Furthermore, Zhang et al. reported that MET may be superior to HIIT in preventing liver cancer development in mice [20]. Therefore, in this study, we used naturally aging mice subjected to either MET or HIIT regimes. Various parameters including cardiac function, myocardial remodeling, myocardial fibrosis, oxidative stress, apoptosis, and aging were analyzed to clarify the exercise training intensity that can prevent cardiac damage in the natural aging process.

## RESULTS

### MET improves myocardial function in aging mice

**Figure 1:**
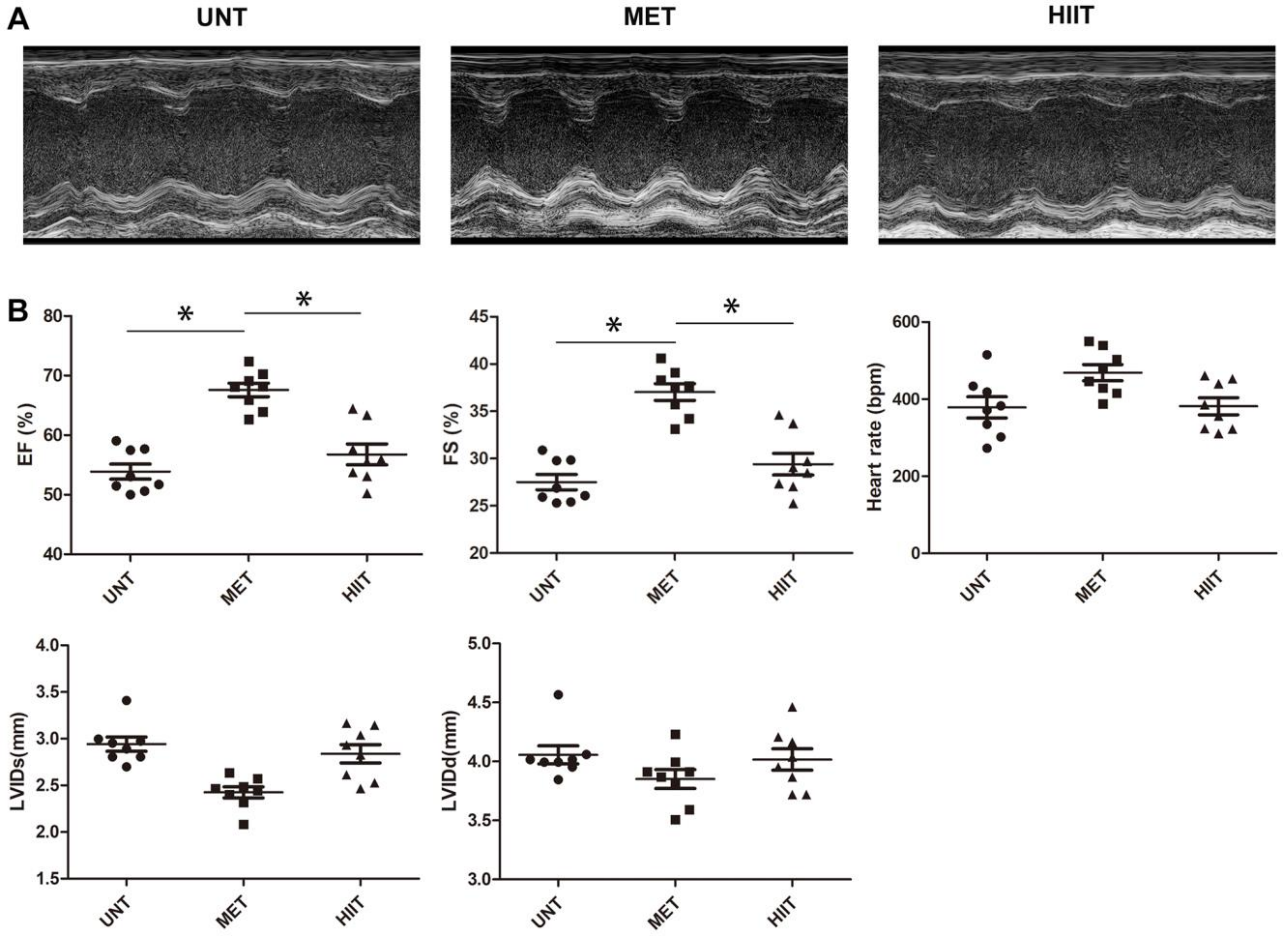
**MET improves myocardial function in aging mice.** (**A**) Representative M-mode echocardiogram. (**B**) LV ejection fraction (EF), LV fraction shortening (FS), LV internal dimension end-systolic (LVIDs), LV internal dimension end-diastolic (LVIDd), and heart rate were evaluated via echocardiography. *n* = 8 mice from each group, ^*^P < 0.05.

To determine whether exercise training protects against cardiac damage, we examined parameters related to cardiac function in mice (Figure 1A). The untrained (UNT) mice exhibited impaired cardiac function, as evidenced by a decrease in the left ventricular rejection fraction and fraction shortening (LVEF and LVFS). These decreases were also observed in the HIIT group, but they were significantly abrogated in the MET group (Figure 1B).

### Metabolic characterization and cardiac tissue damage

**Figure 2:**
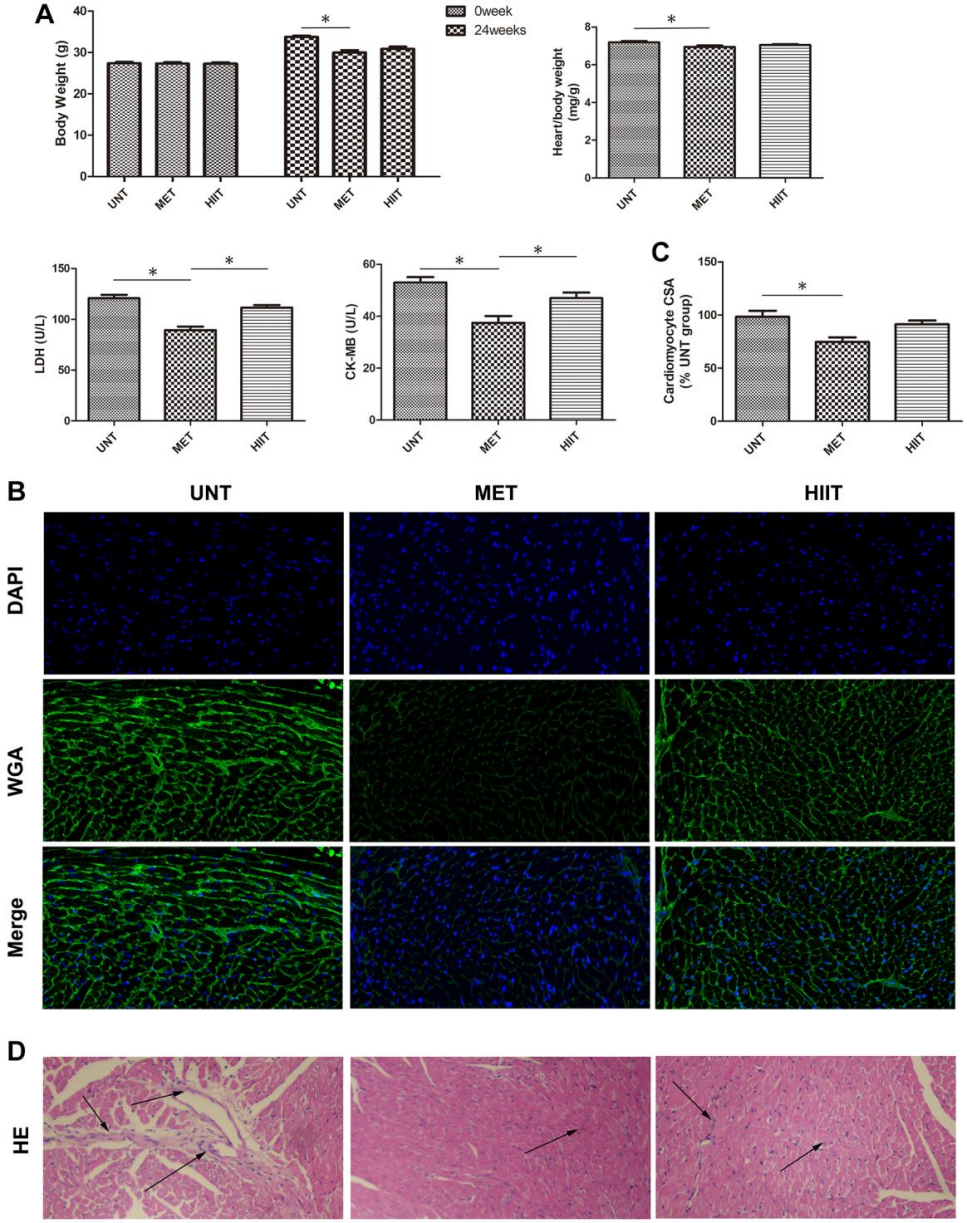
**Metabolic data and cardiac tissue damage in the different groups after exercise training.** (**A**) Quantitative analysis of body weight (0 week and 24 weeks), heart/body weight, LDH, and CK-MB levels in the different groups. *n* = 8 per group. ^*^P < 0.05. (**B**) Representative photomicrographs of myocardium stained with WGA (green fluorescence) and DAPI (blue fluorescence). (**C**) Graph showing the cardiomyocyte CSA. (**D**) H and E staining showing structure damage in cardiac tissue. Magnification 40×. The arrows indicate positively stained cells. *n* = 3 per group. ^*^P < 0.05.

The mice in the UNT group showed considerably increased body weight (24 weeks), heart/body weight, serum lactate dehydrogenase (LDH), creatine kinase MB (CK-MB) levels, but these metrics were significantly decreased in the MET group (Figure 2A). Wheat-germ agglutinin (WGA) and hematoxylin and eosin (H and E) staining revealed cardiomyocyte hypertrophy and inflammatory cell infiltration. Compared with the MET group, the UNT group exhibited myocardial hypertrophy, as evidenced by increased cardiomyocyte cross-sectional area (CSA, Figure 2B–2C) and inflammatory cell infiltration (Figure 2D), both of which could be attenuated by MET.

### MET prevents cardiac remodeling and reduces myocardial fibrosis in aging mice

**Figure 3:**
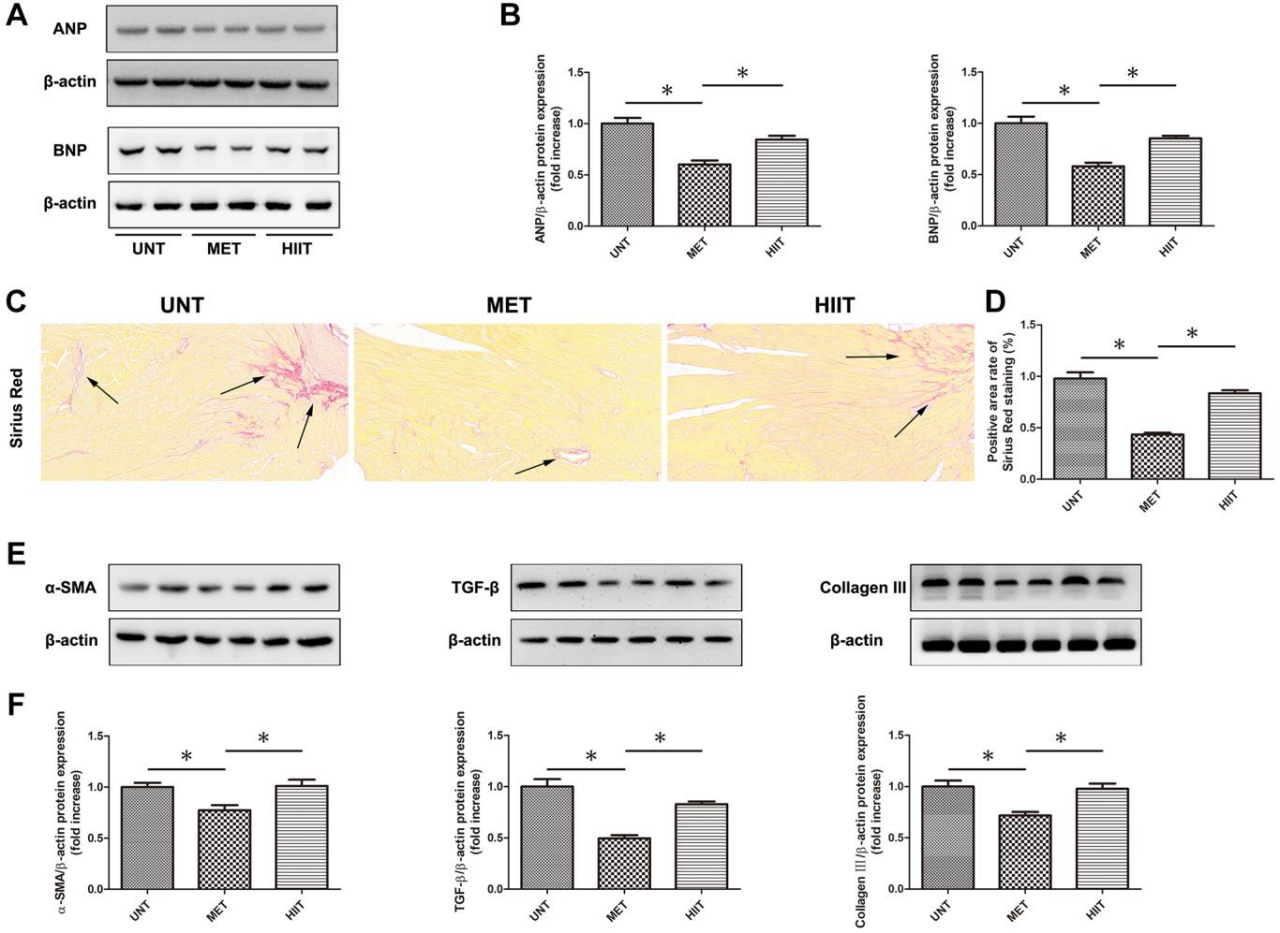
**MET prevents cardiac remodeling and reduces myocardial fibrosis in aging mice.** (**A**) The expression of cardiac remodeling-associated biomarkers, including ANP and BNP. (**B**) Quantification of the relative protein expression levels. (**C**) Sirius Red staining for collagen deposition in the cardiac tissue; (**D**) Quantification of positive staining. (**E**) Cardiac collagen III, α-SMA, and TGF-β levels as assessed by western blotting. (**F**) Quantification of relative protein expression levels. Magnification 40×. The arrows indicate positively stained cells. *n* = 6–8 per group. ^*^P < 0.05.

The levels of cardiac remodeling-associated biomarkers, including ANP and BNP, were significantly increased in the hearts of mice in the UNT group and decreased in the heart of mice in the MET group (Figure 3A–3B). Sirius Red staining was performed to assess cardiac tissue collagen deposition. Aging resulted in distinctive interstitial and perivascular fibrosis in the heart, which was attenuated by MET (Figure 3C–3D). Moreover, we used western blotting (Figure 3E–3F) to evaluate the levels of indicators related to myocardial fibrosis. An upregulation in the expression of collagen III, α-SMA, and TGF-β in cardiac tissues was observed in the UNT group and it was significantly attenuated by MET.

### MET decreases oxidative stress in aging mice

**Figure 4:**
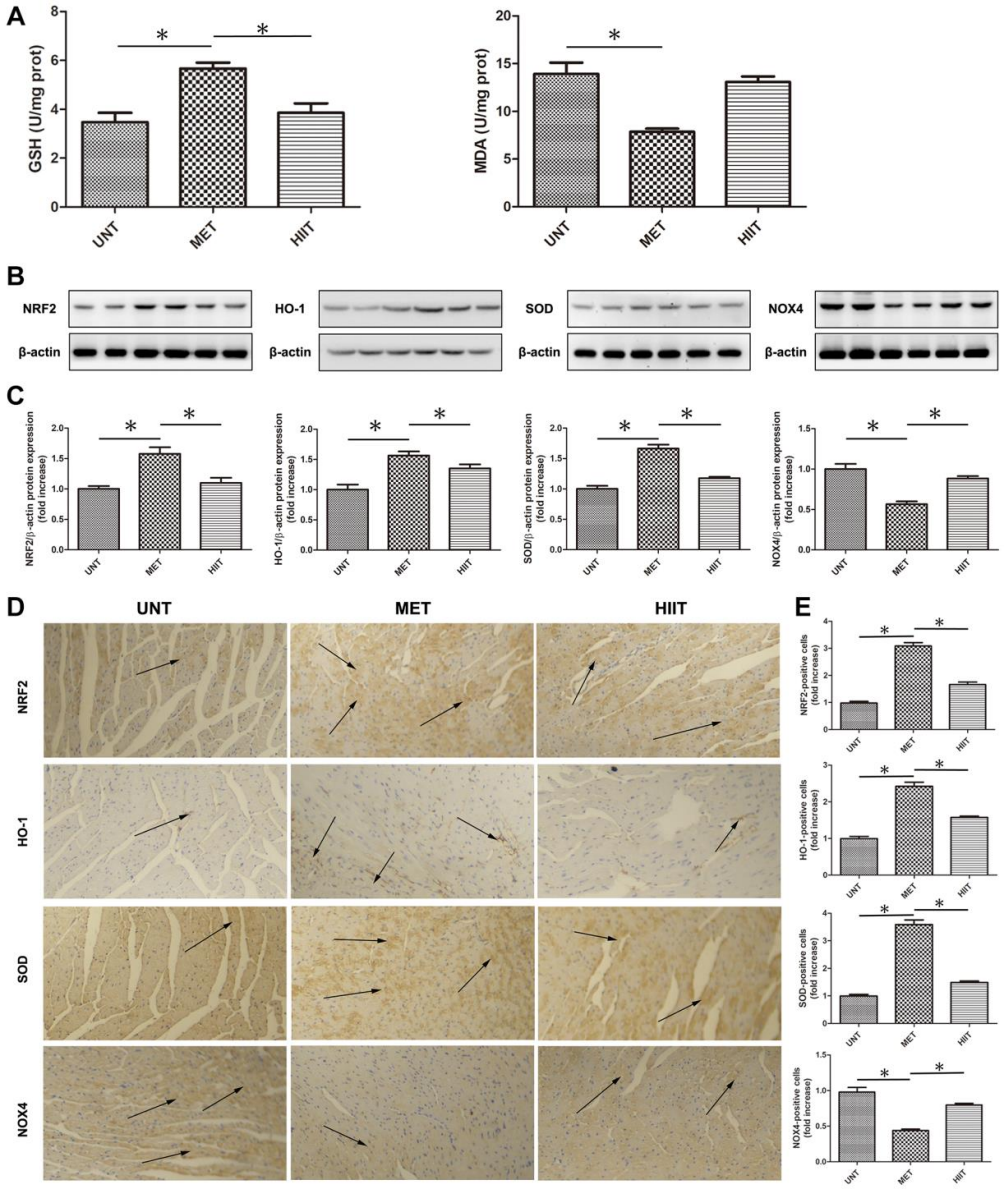
**MET decreases oxidative stress in aging mice.** (**A**) Cardiac MDA and GSH levels were quantified using commercial assay kits. *n* = 8 per group. ^*^P < 0.05. (**B**) Cardiac NRF2, HO-1, SOD, and NOX4 levels assessed by western blotting. (**C**) Quantification of relative protein expression levels. (**D**) Representative immunohistochemistry for NRF2, HO-1, SOD, and NOX4 in cardiac tissues. (**E**) Quantification of positive expression. Magnification 40×. The arrows indicate positively stained cells. *n* = 6–8 per group. ^*^P < 0.05.

The malondialdehyde (MDA) level significantly increased in the UNT mouse heart, but the glutathione (GSH) level decreased compared with MET group mice, however, MET restored the GSH level and decreased the MDA level (Figure 4A). Moreover, we used western blotting (Figure 4B–4C) and immunohistochemistry (Figure 4D–4E) to evaluate the levels of indicators related to oxidative stress. The levels of NRF2, HO-1, and SOD were significantly downregulated in the UNT mouse heart, and MET restored these levels. Furthermore, the expression of NOX4 significantly increased in the UNT group, and it was attenuated by MET.

### MET decreases the levels of natural aging markers in aging mice

**Figure 5:**
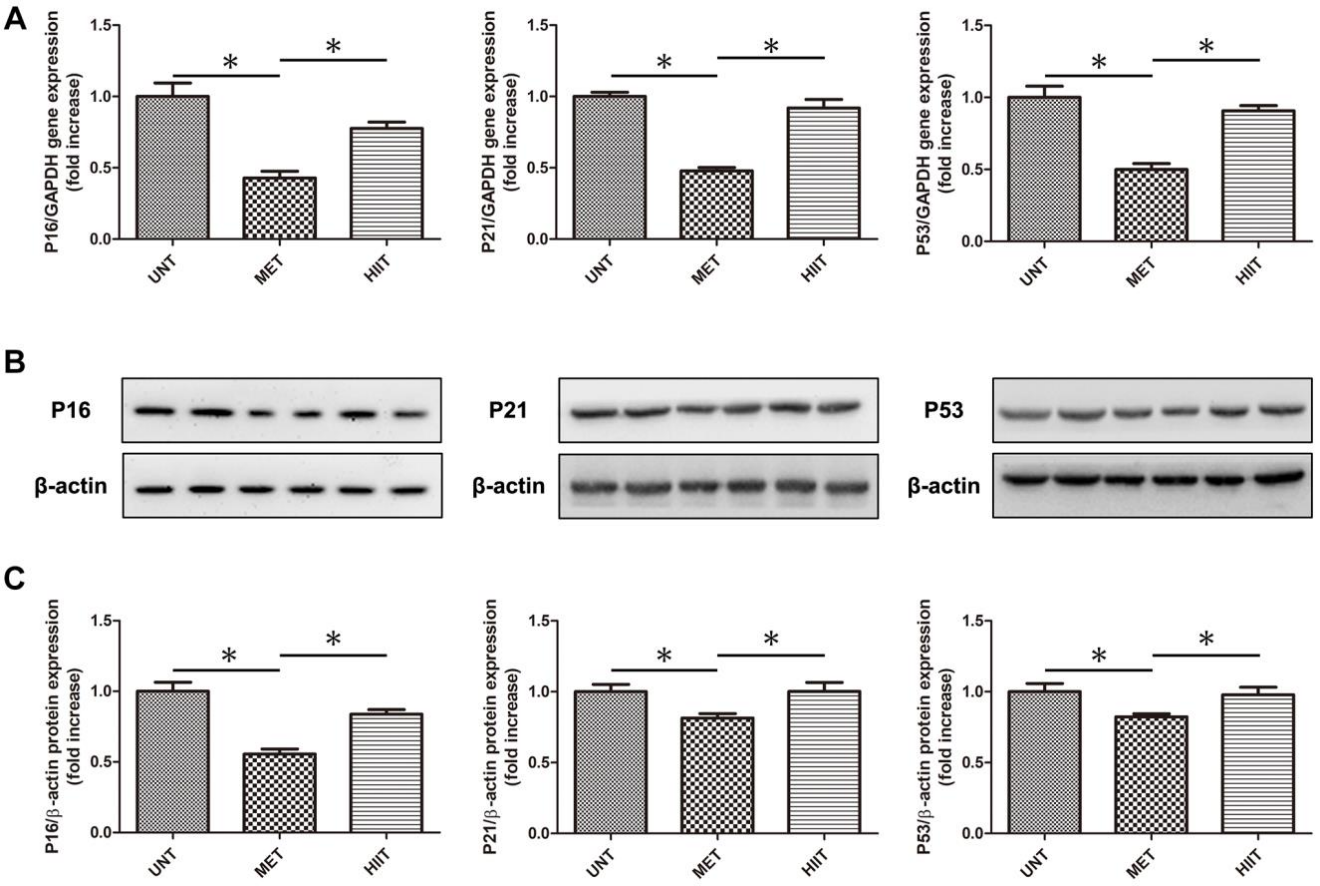
**MET decrease the levels of natural aging markers in aging mice.** (**A**) Cardiac P16, P21, and P53 mRNA levels assessed by real-time PCR. (**B**) Cardiac P16, P21, and P53 protein levels assessed by western blotting. (**C**) Quantification of relative protein expression levels. *n* = 6–8 per group. ^*^P < 0.05.

We evaluated the levels of natural aging markers by real-time PCR and western blotting (Figure 5A–5C). The expression of P16, P21 and P53 significantly increased in the UNT group, and it was attenuated by MET.

### MET decreases apoptosis in aging mice

**Figure 6:**
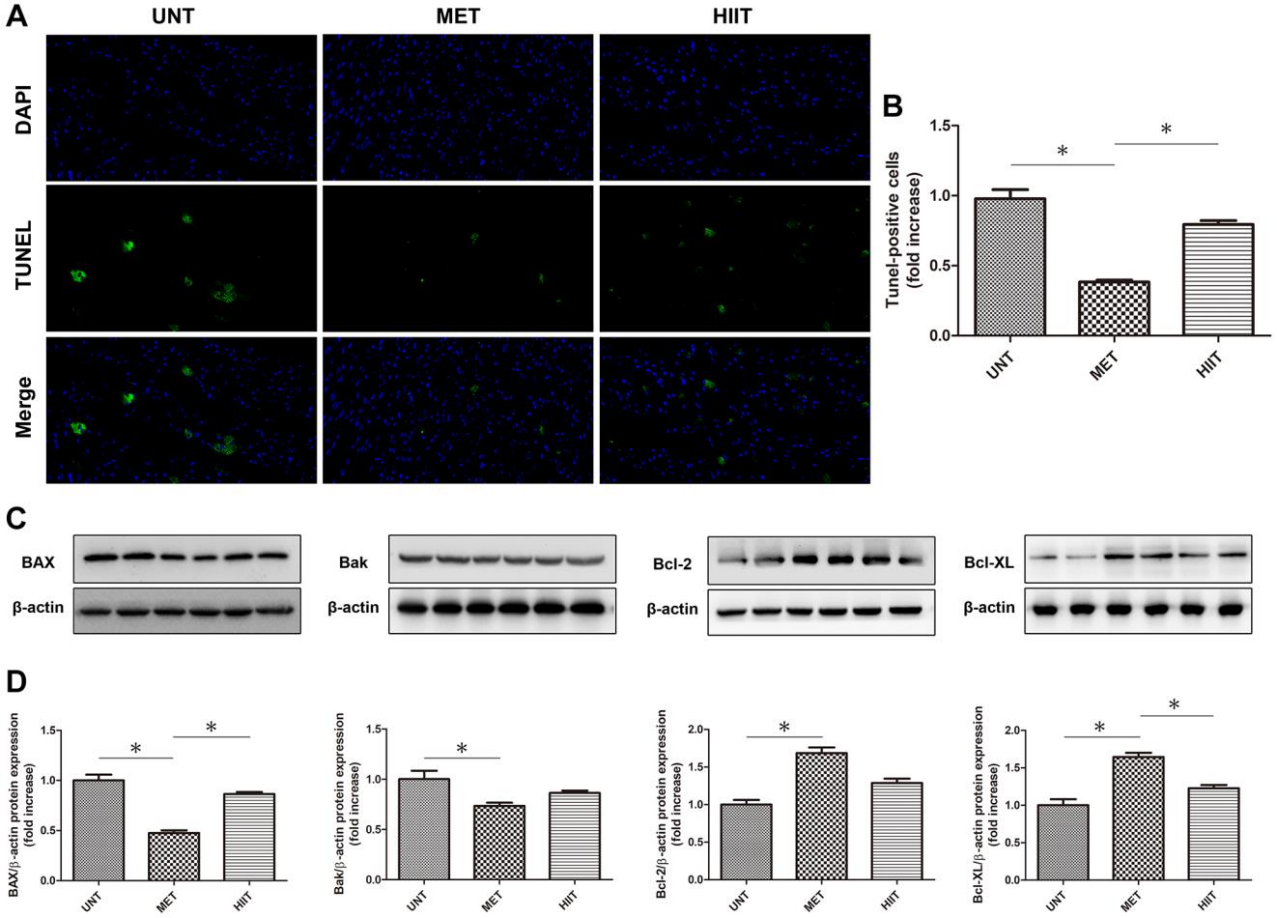
**MET decreases apoptosis in aging mice.** (**A**) TUNEL-stained (green fluorescence) and DAPI-stained (blue fluorescence) photomicrographs. Magnification 40×. (**B**) Quantification of apoptotic cardiomyocytes. (**C**) Cardiac BAX, Bcl-2, Bak, and Bcl-XL levels assessed by western blotting. (**D**) Quantification of relative protein expression levels. *n* = 6–8 per group. ^*^P < 0.05.

The number of TUNEL-positive cells increased in the heart of UNT mice compared with that in the heart of MET mice, whereas cardiac apoptosis was reduced in MET mice (Figure 6A–6B). Immunoblotting (Figure 6C–6D) showed that in the UNT group, the expression of the pro-apoptotic proteins BAX and Bak increased and that of Bcl-2 and Bcl-XL decreased. MET treatment reduced the BAX and Bak levels and enhanced the expression of Bcl-2 and Bcl-XL. These results indicate that MET has a beneficial effect on the heart tissue in aging mice.

## DISCUSSION

**Figure 7:**
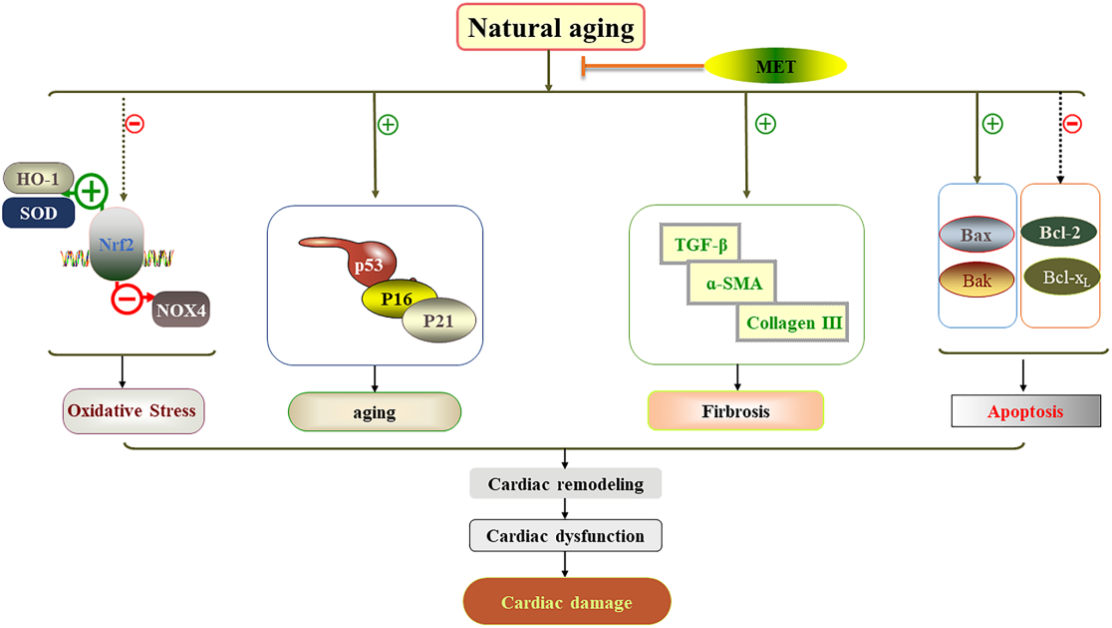
**Schematic diagram showing how MET protects against cardiac damage in aging mice.** MET regimen prevented cardiac dysfunction and reduced cardiac damage by reducing myocardial remodeling, myocardial fibrosis, apoptosis, aging related factor level and oxidative stress in the aging heart.

In this study, we demonstrated in male C57BL/6J mice that 24-week exposure to MET, but not HIIT, reduced the cardiac damage associated with the natural aging process. Further analysis demonstrated that cardiac dysfunction associated with aging was due to the acceleration of myocardial remodeling, myocardial fibrosis, oxidative stress, and apoptosis. Importantly, the MET regimen prevented cardiac dysfunction and reduced cardiac damage. These findings are summarized in Figure 7. However, HIIT had no such effects.

Exercise training is considered beneficial to the cardiovascular system. Regular physical exercise has been shown to improve physical health, improve cardiac function and strengthen the cellular mechanisms that prevent cardiac damage [21–23]. Regular exercise throughout the life reduces cardiovascular diseases and improves functional ability [24]. Studies have shown that voluntary exercise throughout the life can improve age-related gene expression changes in the heart, suggesting that exercise can maintained the heart young [25]. However, the optimal exercise training intensity to protect cardiac damage remains unknown.

Cardiac remodeling and hypertrophy include changes in cell size and morphology [26]. Myocardial remodeling occurs during heart aging, and it involves hypertrophy and fibrosis [7]. To the best of our knowledge, no study has elucidated how different exercise training intensities affect the associated natural aging-induced heart remodeling and fibrosis in mice. The results presented in Figures 2C–2D and 3 show that aging induces cardiac remodeling and fibrosis, furthermore, the results revealed that compared to HIIT, MET effectively reduces myocardial remodeling and fibrosis.

Oxidative stress plays an important role in regulating various physiological and pathogenic processes [27, 28] Previous studies have shown that aging is associated with decreased antioxidant capacity and increased active oxygen accumulation. Moreover, oxidative stress is strongly associated with cardiac hypertrophy and heart failure [29–31]. Studies have also shown that nuclear erythroid-2-p45-related factor-2(Nrf2) plays an important role in regulating most antioxidant and cytoprotective genes in the heart [32, 33]. In the present study, MET upregulated the expression of NRF2 in myocardial tissues, but HIIT did not increase the expression of NRF2. The levels of other antioxidant proteins and metabolites such as HO-1, SOD, and GSH, were also increased; however, the levels of oxidant proteins and metabolites such as NOX4 was found decreased. Shanmugam et al. reported that MET can protect against cardiac damage by enhancing Nrf2-antioxidant signaling and attenuating oxidative stress [34].

Further research is required to elucidate the potential mechanism of MET on the natural aging process. Cell senescence is a characteristic of aging. It is an important contributor to age-related diseases, and the important molecular pathway of senescence is the p16–retinoblastoma protein pathway [35]. The number of senescent cells in an aging heart increases, similar to that in other tissues, and it leads to heart failure. Here, the results revealed that MET, unlike HIIT, attenuates the aging of the heart by downregulating the expression of p16, p21, and p53.

Aging is a complex process of life, characterized by degenerative changes in the aging individual organs and gradual decline in physiological functions [36]. The decrease in the absolute number of myocardial cells is due to increased apoptosis [37]. Our study showed that the number of apoptotic cells increased in naturally aging mice, whereas apoptosis was reduced in the MET group mice. Cui et al. reported that exercise training could attenuate middle-aged maturity-induced cardiac apoptosis [38].

The present study had some limitations. The first limitation is associated with the research design, where in only one exercise duration was investigated. The second limitation, also associated with the study design, is that the animal model does not capture the overall complexity of the processes occurring in humans. Lastly, the samples of mice were without power calculation for group size.

In summary, this study clearly demonstrates the protective effect of MET on cardiac damage in the natural aging process through comprehensive pathways, including improving heart function and attenuating myocardial remodeling, myocardial fibrosis, oxidative stress, and apoptosis. Based on our findings, we propose that MET may be a major method to prevent the cardiac damage induced by natural aging. More clinical studies are needed to clarify the treatment of MET as the prevention of cardiac damage caused by the natural aging of possible therapeutic applications.

## MATERIALS AND METHODS

### Animals

Twenty-four male C57BL/6J mice aged 31 weeks were purchased from Shanghai Model Organisms Center (Shanghai, China) and mice were allowed to adapt to one week. All mice were divided into three groups (eight animals per group) as follows: a UNT control mouse group, an MET group, and a HIIT group. Four mice were housed per cage, and they had access to standard rodent diet and water *ad libitum*. They were maintained under controlled temperature and humidity conditions, under a 12-h light/dark cycle. Our study lasted 24 weeks of the exercise training regimens, after which blood and heart tissue were collected. At the end of the study, the mice were euthanized with a high dose of pentobarbital (100 mg/kg, intraperitoneally), and lack of respiration and heartbeat was used as an indicator of mouse death. Serum was obtained by centrifuging clotted blood collected from the eye sockets of the mice and stored at −80°C. The heart tissue was snap-frozen in liquid nitrogen for mRNA isolation and immunoblotting analyses. All studies involving animal experimentation were followed the National Institutes of Health Guidelines on the Care and Use of Animals.

### Exercise training regimen

Mice in the two exercise training groups were subjected to exercise 5 days per week for 24 consecutive weeks. Under light conditions, the mice were subjected to the following training regimens on a treadmill (No. XR-PT-10B; Shanghai XinRuan Information Technology Co., Ltd. Shanghai, China): MET (50 min/day; 10 meters/min; 7% grade) [32] or HIIT with a 25% grade that contained 10 sets of 2 min high-intensity running (17–19 m/min) sessions, with 2 min of active rest time (8 m/min) in between each session for a total of 40 min [39]. One week before training, the mice were allowed to adapt to the treadmill. The UNT group mice were placed on the track of the treadmill but were not made to exercise for 5 days (50 min/day) per week for 24 consecutive weeks.

### Echocardiography

After 24 weeks of treatment, echocardiography was performed using the Vevo 2100LT micro-ultrasound system (FUJIFILM VisualSonics, Inc., Ontario, Canada). The mice were first anesthetized with 1.5% isoflurane, after which they were immediately placed on a 37°C thermostat to maintain normal body temperature. The position and direction of the ultrasound beam were slowly adjusted to obtain an echocardiogram of the left ventricle. We acquired M-mode images to evaluate left ventricular function parameters.

### Serum measurements

The LDH and CK-MB levels were examined using commercial reagent kits (Nanjing Jiancheng Bioengineering Institute, Nanjing, China).

### Cardiac oxidative stress analysis

Cardiac tissue was homogenized in saline solution at a ratio of 1:9 mg/μL. The homogenate that was spun for 5 min at 7,000 rpm, after which the supernatant was collected and used for GSH and MDA measurements using commercially available kits according to the manufacturer’s directions (Nanjing Jiancheng Bioengineering Institute, Nanjing, China).

### Histological staining

Mice were anesthetized with isoflurane (1.5%) at the beginning, when the hearts were fixed by perfusion with 10% buffered formalin and used isoflurane (4%) via a nozzle placed over the nose. The hearts were fixed overnight at room temperature, transferred into 70% ethanol, and then embedded in paraffin. Paraffin-embedded cardiac tissue slices were deparaffinized via immersion in xylene (three times, 5 min each) rehydrated in a descending alcohol series (100%, 90%, 80%, and 70% alcohol, 5 min each), dehydrated in an ascending series of ethanol (70%, 80%, 90%, and 100% alcohol, 5 min each), and deparaffinized via immersion in xylene (three times, 5 min each). Histological changes were detected by staining 5-μm-thick sections with HE, FITC-conjugated WGA, or Sirius Red stain. Images were acquired microscopically using a B × 40 upright light microscope (Olympus, Tokyo, Japan).

### Immunohistochemistry

The coronal sections of the heart tissues were fixed in 10% formalin, dehydrated in an ascending series of ethanol, and embedded in paraffin for histological evaluation. For immunohistochemical staining, the heart sections were deparaffinized and rehydrated. Next, the sections were blocked with 3% H_2_O_2_ in methanol for 15 min to inactivate endogenous peroxidases and then incubated overnight at 4°C with one of the following primary antibodies: rabbit anti-NOX4, (1:100; Proteintech), rabbit anti-NRF2 (1:200; Proteintech), rabbit anti-HO-1 (1:100; Abcam, Cambridge, UK), and rabbit anti-SOD (1:200; Proteintech). The sections were then incubated for 30 min at room temperature with a goat anti-rabbit HRP secondary antibody (Histofine Simple Stain kit; Nichirei, Tokyo, Japan). All sections were examined with an Olympus B × 40 upright light microscope (Olympus, Tokyo, Japan).

### TUNEL staining

The hearts were embedded in paraffin, and serially sectioned to 5 μm thickness. The sections were deparaffinized and hydrated in xylene and gradient concentrations of ethanol, and then incubated in proteinase K (37°C, 22 min) and stained using the Fluorescein TUNEL Cell Apoptosis Detection kit (Servicebio Technology Co., Ltd., Wuhan, China). All images were captured using a fluorescence microscope (Nikon). The cells that were positive for TUNEL staining and aligned with DAPI staining were considered apoptotic cells and counted.

### Western blotting

Proteins were extracted using radioimmunoprecipitation assay buffer (P0013B; Beyotime, Shanghai, China). The protein samples were first separated by 10% sodium dodecyl sulfate-polyacrylamide gel electrophoresis (SDS-PAGE), and then transferred to polyvinylidene fluoride membranes (Immobilon, Millipore, Billerica, MA, USA). The membranes were blocked with 5% skim milk in TBST buffer (TBS containing 0.1% Tween-20) at room temperature for 1 h, and then incubated with one of the following primary antibodies at 4°C overnight: rabbit anti-ANP (1:500; Invitrogen, USA), rabbit anti-BNP (1:500; Invitrogen), rabbit anti-collagen III (1:1000; Proteintech), rabbit anti-α-SMA (1:2000; Proteintech), anti-TGF-β (1:1000; Proteintech), rabbit anti-NOX4 (1:1000; Proteintech), rabbit anti-NRF2 (1:500; Proteintech), rabbit anti-HO-1 (1:1000; Abcam, England), rabbit anti-SOD (1:2000; Proteintech), rabbit anti-P16 (1:500; Proteintech), rabbit anti-P21 (1:1000; Proteintech), rabbit anti-P53 (1:1000; Proteintech), rabbit anti-BAX (1:5000; Proteintech), rabbit anti-Bcl-2 (1:2000; Proteintech), rabbit anti-Bak (1:1000; Proteintech), rabbit anti-Bcl-XL (1:2000; Proteintech), and rabbit anti-β-actin (1:1000; Proteintech). After washing, the membranes were incubated with the appropriate secondary antibody (anti-rabbit Ig-G, 1:2000; Proteintech) for 1 h. The immunoreactive proteins were quantified using NIH ImageJ software. β-Actin was used as an internal control. The protein levels are expressed as protein/β-actin ratios.

### RNA isolation and real-time PCR (qPCR)

Total RNA was isolated from cardiac tissues and complementary DNA (cDNA) was synthesized using the TransScript One-Step gDNA Removal and cDNA Synthesis SuperMix kit according to the manufacturer’s protocol. Gene expression was quantitatively analyzed using qPCR and the TransStart Top Green qPCR SuperMix kit. GAPDH was amplified and quantitated in each reaction to normalize the relative amounts of the target genes. Primer sequences are listed as follow: P16: forward (5′-TCGTGCGATATTTGCGTTCC-3′) and reverse (5′-GGATTGGCCGCGAAGTT-3′); P21: forward (5′-CAGCCATGACGAGCTGTTCT-3′) and reverse (5′-CTTTCGGTACCTTCGCCCTC-3′); P53: forward (5′-CCCTCTGAGCCAGGAGACATT-3′) and reverse (5′-CCCAGGTGGAAGCCATAGTTG-3′); GAPDH: forward (5′-CCTCGTCCCGTAGACAAAATG-3′) and reverse (5′-TGAGGTCAATGAAGGGGTCGT-3′).

### Statistical analyses

All analyses were performed using the Statistical Package for Social Sciences version 23.0 (SPSS, Chicago, IL, USA). The data were expressed as mean ± SEM. Statistical differences among multiple groups of data were analyzed using the one-way analysis of variance (ANOVA) with a pos-hoc Tukey’s test. In all statistical comparisons, a *p*-value < 0.05 was used to indicate a statistically significant difference.
